# Synergistic Effects of Sm and C Co-Doped Mixed Phase Crystalline TiO_2_ for Visible Light Photocatalytic Activity

**DOI:** 10.3390/ma10020209

**Published:** 2017-02-21

**Authors:** Fuchang Peng, Honglin Gao, Genlin Zhang, Zhongqi Zhu, Jin Zhang, Qingju Liu

**Affiliations:** 1 School of Materials Science and Engineering, Yunnan Key Laboratory for Micro/Nano Materials & Technology, Yunnan University, Kunming 650091, China; Pzhupfc@163.com (F.P.); genlinzhang@126.com (G.Z.); zhuzhongqi@ynu.edu.cn (Z.Z.); zhj@ynu.edu.cn (J.Z.); 2 College of Material Engineering, Panzhihua University, Panzhihua 617000, China

**Keywords:** mixed phase TiO_2_, co-doped TiO_2_, synergistic effects, sol-gel, photocatalytic activity

## Abstract

Mixed phase TiO_2_ nanoparticles with element doping by Sm and C were prepared via a facile sol-gel procedure. The UV-Vis light-diffuse reflectance spectroscopy analysis showed that the absorption region of co-doped TiO_2_ was shifted to the visible-light region, which was attributed to incorporation of samarium and carbon into the TiO_2_ lattice during high-temperature reaction. Samarium effectively decreased the anatase-rutile phase transformation. The grain size can be controlled by Sm doping to achieve a large specific surface area useful for the enhancement of photocatalytic activity. The photocatalytic activities under visible light irradiation were evaluated by photocatalytic degradation of methylene blue (MB). The degradation rate of MB over the Sm-C co-doped TiO_2_ sample was the best. Additionally, first-order apparent rate constants increased by about 4.3 times compared to that of commercial Degusssa P25 under the same experimental conditions. Using different types of scavengers, the results indicated that the electrons, holes, and •OH radicals are the main active species for the MB degradation. The high visible-light photocatalytic activity was attributed to low recombination of the photo-generated electrons and holes which originated from the synergistic effect of the co-doped ions and the heterostructure.

## 1. Introduction

Recently, the preparation and characterization of titanium oxide (TiO_2_) nanopowders have been intensely investigated for applications in air cleaning, sensors, solar cell, gene therapy, and photocatalytic water splitting because of their chemical stability against photocorrosion and chemical corrosion, nontoxicity, and cost-effectiveness [1,2,3]. Although TiO_2_ generally shows high activity for the photocatalytic oxidation of organic pollutants [1], more widespread applications of TiO_2_ as a photocatalyst have been limited due toits low use of solar energy (only active in the ultraviolet region) and its relatively high recombination rate between the photo-generated electrons and holes [4]. To increase the photocatalytic efficiency of TiO_2_, various methods have been used to enhance its absorption of the solar energy and to inhibit the recombination of photogenerated electron-hole pairs. A prominent approach is to dope TiO_2_ with transition metals or nonmetallic elements. Metal ion dopants, such as Fe [5], V [6], Bi [7], and Sm [8], can act as electron or hole traps and, consequently, decrease the electro-hole pair recombination rate. Indeed, special efforts have been dedicated to studying TiO_2_ doped with metal ions. Among metal ions, rare-earth metals often serve as catalyst or promote catalytic properties due to their incompletely occupied 4f and empty 5d orbitals. In particular, Sm [8,9,10] has received much attention due to its high efficiency in improving photocatalytic activity as well as its low price. Torres et al. have shown that Sm^3+^ doped TiO_2_ exhibits enhanced photocatalytic activity under sunlight degradation of diuron herbicide compared to that of TiO_2_ [9]. Deng et al. have prepared mesoporous Sm-TiO_2_, and showed that the Sm^3+^ doping enhanced the photocatalytic activity of TiO_2_ in the elimination of gaseous acetone and methanol [10]. Meanwhile, the doping of some non-metal ions, such as N [11], C [12], S [13], or B [14] also seems to be efficient for improving the photocatalytic activity of TiO_2_. Furthermore, co-doped TiO_2_ with two or more ions has been investigated and has attracted much attention. The theoretical and experimental results indicated that the photocatalytic activity of TiO_2_ co-doped with more than one ion is better than that of the TiO_2_ doped with a single ion, because of the synergistic effect of co-doped ions [15,16]. Huang et al. have synthesized Sm and N co-doped TiO_2_ and demonstrated that the co-doped photocatalysts have better activity than that of N doped TiO_2_ in the degradation of 4-chlorophenol [17]. However, there is no report on the co-doping of C and Sm ions into the TiO_2_ lattice.

The heterostructure between the anatase and rutile phases of TiO_2_ was demonstrated to be efficacious in controlling the photo-generated charge migration across the heterojunction interface and enhancing the charge separation [18,19,20,21,22,23,24]. Thus the mixed phase TiO_2_ exhibits higher photocatalytic activity than that of the individual composition. Among the reported TiO_2_ materials, it has been shown in numerous studies that there is a positive interaction of anatase and rutile TiO_2_ particles in Degussa P25, which is widely accepted as the benchmark. The synergistic effect between the two phases is responsible for enhancing the electron-hole separation, thus increasing the photocatalytic activity [22,23,24]. Indeed, experimental evidence has revealed that the effect was related to the relative Fermi levels and shapes of anatase and rutile particles, indicating that the charge migration between the mixed phases is dependent on the experimental conditions [25,26,27].

In this paper, an anatase/rutile mixed phase of TiO_2_ materials with co-doping of samarium and carbon was prepared via an ordinary sol-gel method. The anatase/rutile ratio of the material can be tuned by varying the dopants. The microstructure and physicochemical characteristics were characterized by X-ray diffraction (XRD), Transmission electron microscope (TEM), Fourier transform infrared (FTIR) spectra, X-ray photo electron spectroscopy (XPS), ultraviolet-visible (UV-Vis) spectra and photoluminescence (PL). The photocatalytic activities for the degradation of MB under visible-light irradiation (30 W fluorescent lamp) were evaluated. The synergistic effects of the co-doping and heterojunction on photocatalytic activity were also studied.

## 2. Results

**Figure 1:**
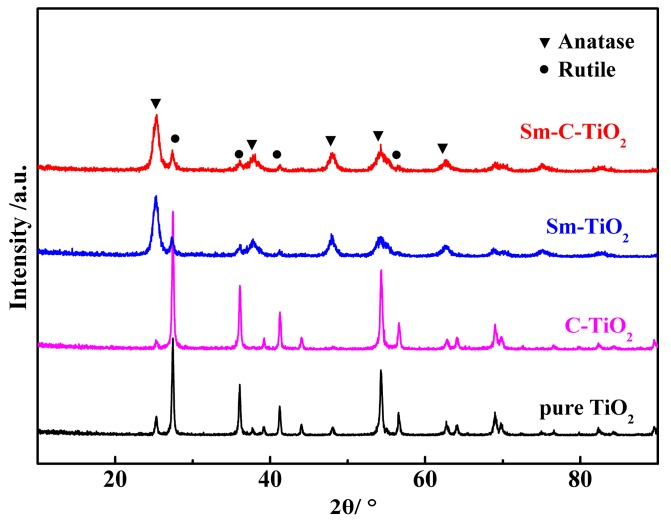
XRD patterns of the pure TiO_2_, C-TiO_2_, Sm-TiO_2_, and Sm-C-TiO_2_ samples calcined at 500 °C.

XRD was used to investigate the crystal structure changes after introduction of the dopants into the TiO_2_, and the patterns are shown in Figure 1. Viewed from the figure, the XRD pattern of undoped TiO_2_ can be indexed to the anatase TiO_2_ (JCPDS No. 21-1272) and rutile TiO_2_ (JCPDS No. 88-1175), where the anatase phase percentage is 12.6%. Furthermore, all diffraction lines of the entire sample can be assigned for both the anatase and rutile phases, and no samarium oxide-related diffraction line was observed. Average crystallite sizes of the TiO_2_ samples were measured from X-ray line broadening analysis using the well-known Scherrer equation:

*d* = 0.89λ/*B*cosθ (1)

where *B* is the full-width at half maximum (FWHM) in radians, λ is the wavelength of the X-rays in nanometers (1.5406 Å), θ is the angle between the incident and diffracted beams in degrees, and *d* is the average crystallite size of the powder sample in nanometers [27]. The crystallite size results and crystallographic phases are listed in Table 1. Obviously, the ratio of anatase-rutile phases depends on the dopant species. When C was added to TiO_2_, the percentage of anatase TiO_2_ decreased to 4%. For the TiO_2_ doped with Sm, the anatase phase became the dominant phase, with 74.3%. For the C-Sm co-doped TiO_2_, the anatase phase had a slight reduction to 70%, with 30% rutile phase. This result indicates that the C can increase the formation of the rutile phase but Sm can drive the maintenance of the anatase phase.

To further study the effect of samarium doping on the TiO_2_ lattice, the d-spacings of the anatase (101) and rutile (110) diffraction lines were calculated (Table 1). The *d* values of the anatase (101) and rutile (110) plane of the C-TiO_2_ sample remained the same compared to the undoped TiO_2_, indicating that the carbon doping content may not be high enough to induce a change of the d-spacing. When samarium was introduced into the TiO_2_ crystal, a shift of the *d* value to a larger value was observed (Table 1). The substitution of samarium ions for titanium ions in the lattice should be responsible for the observed increase in d-spacings, since the samarium ion has a larger ionic radius than that of titanium (Sm^3+^ = 0.96 Å vs. Ti^4+^ = 0.75 Å) [28]. The average grain size of all samples is also displayed in Table 1. The introduced dopants decreased the size, suggesting more reaction sites on the materials.

**Figure 2:**
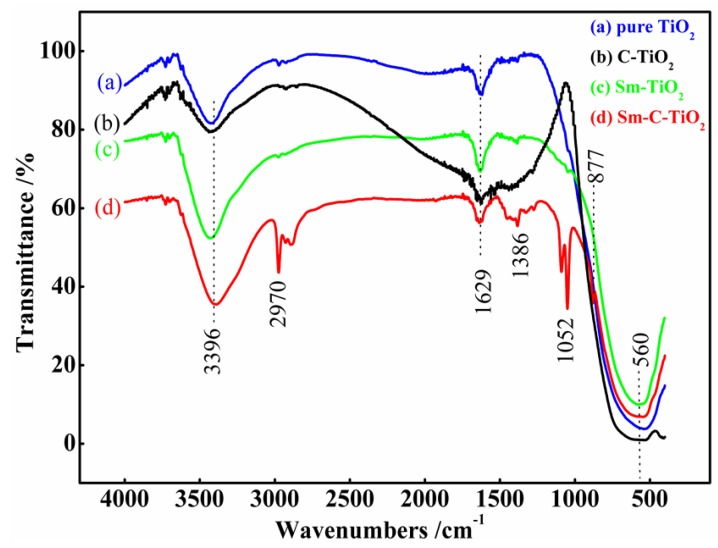
FT-IR spectra of Sm-C-TiO_2_, C-TiO_2_, Sm-TiO_2_, and TiO_2_ samples.

Figure 2 shows the FTIR spectra of samples with different dopants. All spectra show a broad band around 800–400 cm^−1^ that can be assigned to the vibrations of the Ti–O bonds in the anatase and rutile phases of TiO_2_. The stronger peaks at 1629 and 3396 cm^−1^ are attributed to the bending and stretching vibrations of O–H, respectively [17]. Furthermore, three new peaks at 1052, 1386, and 2970 cm^−1^ arise for the co-doped TiO_2_ samples, which can be assigned to C–O stretching vibrations [30], symmetrical vibration of the carboxyl [31], and –CH_3_ stretching vibration [32], respectively. The results of the infrared spectra indicated that the samarium ions coordinated with organic acid that may have remained on the surface of the particles. It can be induced that the samarium dopant is the main active site for the adsorption of MB. Additionally, a shoulder on the main absorption peak appears at about 877 cm^−1^ for co-doped TiO_2_, which is derived from Sm–O stretching vibrations [33], indicating that the carbon introduction drove the Sm ions onto the surface of the TiO_2_.

**Figure 3:**
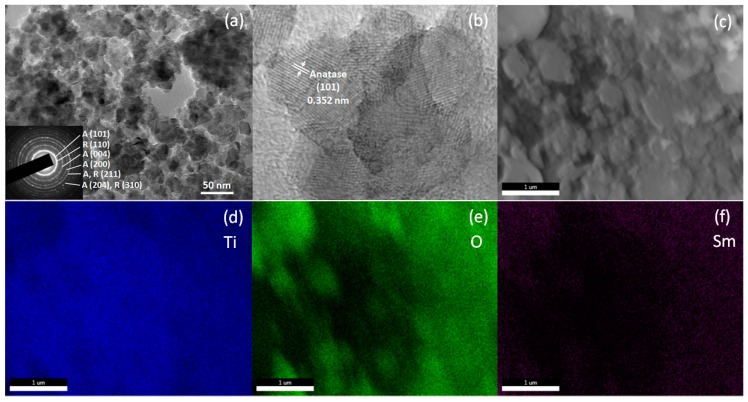
Bright field TEM (**a**); HRTEM (**b**); and SEM image (**c**) of Sm-C-TiO_2_ with the corresponding elemental distribution maps (**d**–**f**). The inset shown in (**a**) is the diffraction pattern of the particles.

In order to further reveal the morphology and microstructure of the samarium and carbon co-doped TiO_2_, the TEM/SAED (selected area electron diffraction) and SEM/EDX (energy-dispersive X-ray spectroscopy) were used to carry out the investigation (Figure 3). Figure 3a shows that the sample was of nanoparticle dimensions with a mean size of 20–60 nm. In the inset of Figure 3a, (SAED) from a group of grains corresponding to the (101), (004), (200),(211), and (204) lattice spacings of the anatase phase, and to the (110), (211), and (310) lattice spacings of the rutile phase, indicated the coexistence of both phases in this sample. Furthermore, the high resolution transmission electron microscopy (HRTEM) image in Figure 3b shows lattice fringes with 0.352 nm spacing, which can be indexed as the (101) lattice spacing of the anatase phase. These results also indicated that the anatase is the dominant phase, which is consistent with the XRD results. No individual samarium oxide species were observed during analysis, which further suggests the incorporation of samarium into the TiO_2_ lattice. Furthermore, the chemical compositions were investigated by EDX, and the corresponding elemental distribution maps are shown in Figure 3d–f. The maps of Ti, O, and Sm were well-defined with sharp (Figure 3c) contrast. The profile of Sm was close to that of Ti and O, which indicated that the Sm is homogeneously and densely distributed throughout the materials.

**Figure 4:**
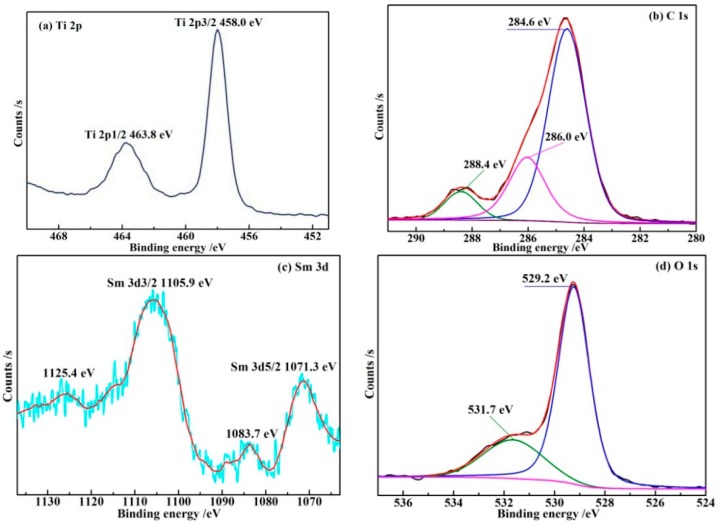
XPS spectra for the Sm-C-TiO_2_. (**a**) Ti 2p; (**b**) C 1s; (**c**) Sm 3d; (**d**) O 1s.

The surface elemental character of the chemical bonds of Sm–C–TiO_2_ was investigated by X-ray photoelectron spectroscopy (XPS), and the results of the high-resolution Ti 2p, C 1s, Sm 3d, and O 1s XPS spectra are shown in Figure 4. The high resolution Ti 2p XPS spectrum consists of two peaks at 458.0 eV and 463.8 eV (Figure 4a), which are assigned to the binding energy of Ti 2p3/2 and Ti 2p1/2, respectively, indicating that the titanium element exists in the form of Ti^4+^ in the Sm-C-TiO_2_ samples. Moreover, the higher binding energies than that of Ti^4+^ in TiO_2_ (453.9 eV and 460.0 eV) suggest a decrease in the electron charge density of the Ti^4+^ ion after the carbon and samarium were inserted.

The carbon species display three bonding states at 284.6, 286.0, and 288.4 eV (Figure 4b). Generally, the C 1s peak at 284.6 eV is assigned to the presence of sp2 type carbon [34]. In addition, the other two C 1s peaks appearing around 286.0 eV and 288.4 eV were ascribed to the oxygen bound species C–O and C=O, respectively, demonstrating that carbon was incorporated into the TiO_2_ lattice by substituting some of the lattice titanium atoms to form the Ti–O–C structure [10,35], and existed in the form of CO_3_^2−^ [36,37], which strongly suggested that the existence of C-doping from the binding energies of the electrons relates to C 1s [38,39]. In the Sm 3d spectra of Sm-C-TiO_2_ (Figure 4c), the Sm 3d5/2 peak appears at 1083.7 eV for the Sm^3+^ states which is in accordance with that of the binding energy in Sm_2_O_3_, and at 1071.3 eV for the Sm^2+^ states [40], and the peak intensity ratios of Sm^2+^/Sm^3+^ is about 8. The Sm 3d3/2 peaks also supported these results [41].

The O 1s core level peak can be composed of two species. Firstly, the peak located at a binding energy of 529.2 eV (Figure 4d) can be assigned to the ionic oxygen in the lattice. Another small shoulder peak situated at 531.7 eV can be associated with adsorbed OH^−^ species, O^−^ species, or oxygen vacancies [20]. Latter type oxygen species have always been regarded as the most active intermediates to oxidize organic pollutants [42].

**Figure 5:**
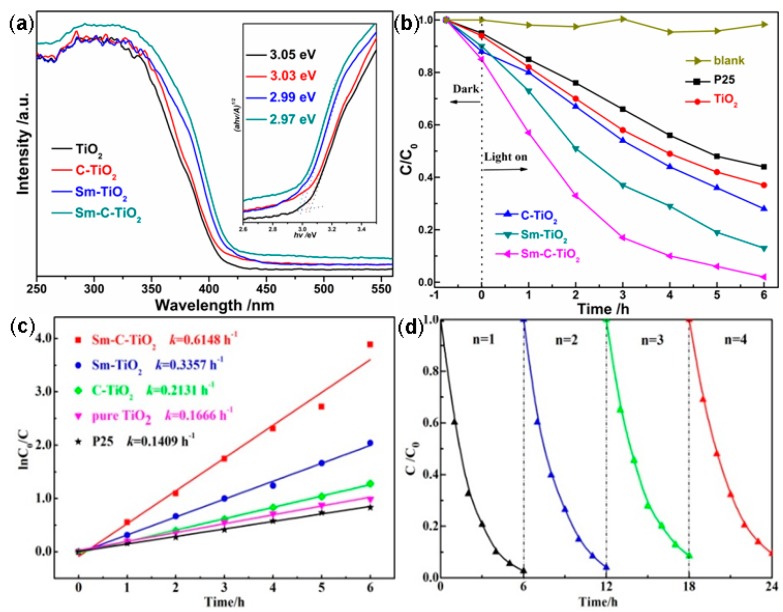
(**a**) Diffusive reflectance UV-Visible absorption profiles of the samples of pure TiO_2_, C-TiO_2_, Sm-TiO_2_, and Sm-C-TiO_2_, inset shows the corresponding Tauc plots as well as the optical band gap values for the undoped and doped TiO_2_. The time course (**b**) and kinetic curves (**c**) of MB photodegradation over different samples under visible light irradiation.(P25; pure TiO_2_; Sm-TiO_2_; C-TiO_2_; Sm-C-TiO_2_). (**d**) Cycling runs in photocatalytic degradation of MB in the presence of Sm-C-TiO_2_ photocatalysts under visible light irradiation.

For a given photocatalyst, the absorption band will be changed when extrinsic dopants go into the lattice. Thus, UV-Vis diffusive reflectance spectroscopy was employed to estimate the band gap energies of the doped TiO_2_ in this investigation. Figure 5a presents the UV-Vis absorption spectra of the prepared undoped TiO_2_ as well as the sample doped with C, Sm, Sm-C, respectively. For a crystalline semiconductor, the optical band gap (*Eg*) can be well estimated from the Tauc’s plots (inset of Figure 5a), which are calculated by using αhυ = A(hυ − *Eg*)*^n^*^/2^, where α is the absorption coefficient near the absorption edge, υ is the frequency of the light (s^−1^), h is Plank’s constant (=6.626 × 10^−34^ J·s), *Eg* is the absorption band gap energy, *n* is decided by the characteristics of the transition in the semiconductor; *n* = 1 as indirect absorption and *n* = 4 as direct absorption [43]. In this case, the mixed phase TiO_2_ can be attributed to an indirect bandgap semiconductor. When the value of the *y* axis ((αhυ)^1/2^ = 0) is zero, an approximation of the band gap energy of the samples can be obtained by the intercept of the tangent to the *x* axis. The results suggested that the band gaps of the as-prepared samples were estimated to be 3.03 eV (for C-TiO_2_), 2.99 eV (for Sm-TiO_2_), 2.97 eV (for Sm-C-TiO_2_), and 3.05 eV (for undoped TiO_2_), as shown in the inset of Figure 5a. From Figure 5a, the undoped TiO_2_ reveals a cutoff wavelength around 400 nm as expected in the UV range, corresponding to the band gap of 3.1 eV, which is consistent with the approximate values obtained from the Tauc plot as well as the band gap values reported previously [44]. All doped TiO_2_ samples exhibited a distinct red shift of the adsorption edges as compared to undoped TiO_2_. A red shift of this type can be attributed to the charge-transfer transition between the dopant (f electrons of samarium and/or p electrons of carbon) and the TiO_2_ conduction or valence band [8,41]. As a result, the samarium and carbon co-doping TiO_2_ can absorb lower photoenergy to a light with a wavelength of 420 nm and thus enhance the photocatalytic activity under visible light irradiation.

The methylene blue (MB) degradation over the undoped and doped TiO_2_ samples was used to evaluate the photocatalytic activity for the investigated samples and the results are shown in Figure 5. In the blank experiment, MB photo-degradation is not observable in the absence of catalyst, which indicates that MB is stable under light illumination. From Figure 5b, it can be seen that after 6 h of visible light illumination, MB removal over undoped TiO_2_ is 62%, which is slightly higher than that of P25. All doped TiO_2_ catalysts showed better photocatalytic activity than undoped TiO_2_. Significantly, samarium and carbon co-doped TiO_2_ catalysts displayed a sharp increase in the photocatalytic activity for MB decomposition, which induced 98% degradation within 6 h of light irradiation. The adsorption ratio of MB which were adsorbed on the surface of TiO_2_, C-TiO_2_, Sm-TiO_2_, and Sm-C-TiO_2_ before the photoreaction are 6%, 12%, 10%, and 14%, respectively. After light irradiation for 6 h, the percentages of photodegraded MB are 62%, 72%, 87%, and 98% over the above samples, respectively, indicating that samarium and carbon dopant may promote the transfer and separation of photo-generated electrons and holes, and then increase the catalytic activity.

The kinetics of the MB photodegradation on the undoped and doped TiO_2_ can be simulated by the pseudo-first-order kinetic equation [45]. Figure 5c presents the linear relationship between ln(*C*_0_/*C*) and the irradiation time. *k* is determined from the slope of the line when ln(*C*_0_/*C*) is plotted versus the corresponding irradiation time and thus the value of *k* gives an indication of the activity of the photo-catalyst. Apparently, for the MB photodegradation, the doped TiO_2_ exhibited the rate constants of 0.615 h^−1^ for the Sm-C co-doping, 0.336 h^−1^ for the Sm doping, and 0.213 h^–1^ for the C doping, which are 4, 2, and 1.3 times higher than 0.166 h^−1^ for the undoped TiO_2_, respectively. This evidence confirms that the rate constants of the photodegrading MB are increased by the doping of carbon and samarium.

Moreover, the photocatalytic stability is always regarded as another important factor for a photocatalyst besides activity [46]. Thus, to evaluate the reusability of the as-prepared Sm-C-TiO_2_ sample, a cycling test was performed repetitively for four cycles for the degradation of MB as shown in Figure 5d. It is revealed that the degradation degree of MB decreases little with increasing recycling runs, indicating a relatively stable photocatalysis with no significant decrease in activity. Notably, it can be concluded that these co-doped TiO_2_ are good photocatalysts for the application of organic pollutant degradation.

**Figure 6:**
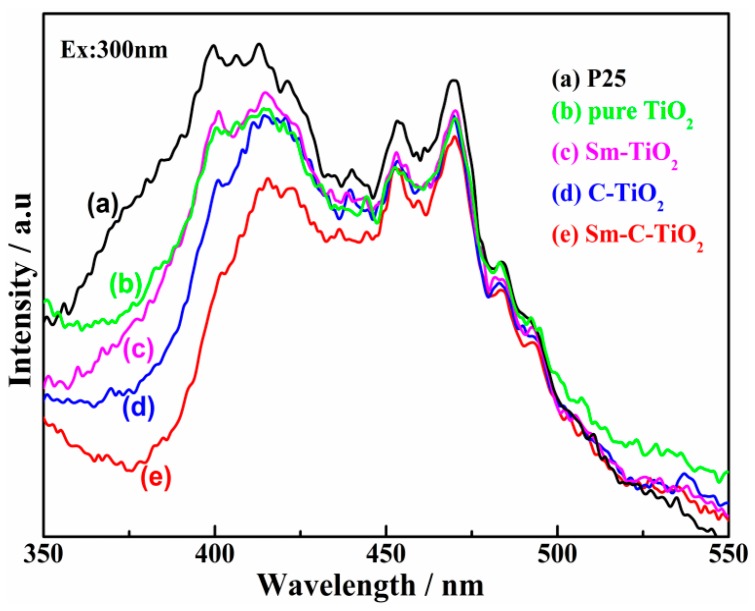
PL spectra of the samples. (**a**) P25; (**b**) pure TiO_2_; (**c**) Sm-TiO_2_; (**d**) C-TiO_2_; (**e**) Sm-C-TiO_2_.

To further disclose the efficiency of charge carrier trapping, immigration, and transfer in the as-prepared samples, photoluminescence measurement was employed as a useful technique to study the surface structure and excited states and to follow the surface processes involving the electron/hole lifetime of TiO_2_ [47]. In general, the photoluminescence emissions (PL) on semiconductor materials originated from the radiative recombination of photo-generated electrons and holes, and the PL signals could be excited by using two major photo-physical processes [48]. The first is the direct band-band transition photoluminescence, which is the release of energy as PL radiation alongside that of the photo-generated electrons, which can transfer from the conduction band to the valance band after the irradiation by light greater than the band gap energy. Another is the illumination of the vacancy or defect state, whose process is the excited electrons firstly transferred from the conduction band to vacancies or defects via non-radiative transition, and subsequently transferred to the valance band via radiative transition with the release of PL signals. In this investigation, the room-temperature PL spectra of undoped and doped TiO_2_ materials (Figure 6) show broad emissions between 550 and 375 nm. Both types of emissions are simultaneously present on all TiO_2_ samples at room temperature and are affected by surface chemistry [49]. A broad and intensive emission in the 350–430 nm range has been attributed to the recombination of photo-generated electrons and holes in the mixed phase TiO_2_ crystallite. Such a recombination process was less pronounced in the codoped TiO_2_, indicating that the recombination was suppressed for C and Sm co-doped into TiO_2_. Besides, multiple PL signals in the visible region (430–550 nm) are apparent for all samples, which corresponded to the shallow traps on the surface or bulk defect sites [50,51]. The carbon and samarium weakly reduced the PL intensity which originates from the increase of octahedral dipole moments in TiO_2_ due to the changes of the lattice parameters. This may also facilitate the separation of photo-generated electron-hole pairs, thus enhancing the photocatalytic activity.

**Figure 7:**
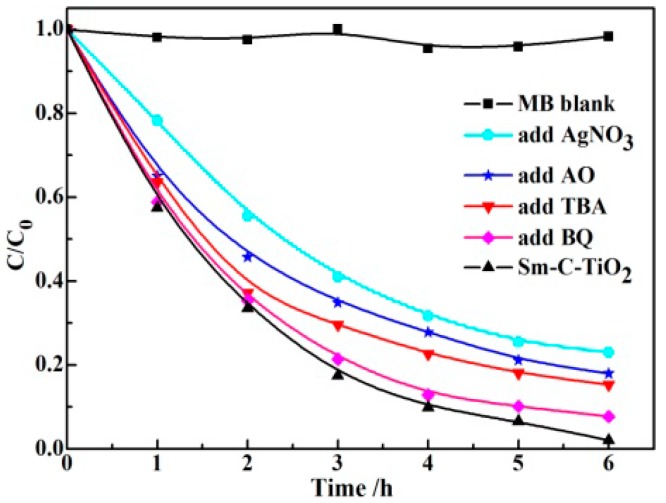
Photocatalytic degradation of MB over Sm-C-TiO_2_ samples under visible light irradiation with the addition of different scavengers: MB blank, BQ, TBA, AO, and AgNO_3_.

In order to investigate the role of the main active species such as holes, electrons, hydroxyl radicals (•OH), and superoxide radicals (•O_2_^–^), different types of active species scavengers were used [52]. Figure 7 shows the photocatalytic activities of Sm-C-TiO_2_ in the degradation of MB under conditions of adding different scavengers. Without the addition of scavengers, the photocatalytic conversion ratio (PCR) of the main absorption peak of MB on TiO_2_ was 98% after 6 h of irradiation. Among the photocatalytic processes, the separation and transfer of electrons and holes in the semiconductor are the most important factors. Therefore, to investigate the mechanism of the process, AgNO_3_ as an electron scavenger was first employed to determine the influence of the specific reactive species of the electron. When 0.1 g of AgNO_3_ was added, the PCR was reduced to 72% after 6 h of irradiation. The decrease of the amount of electrons reduced the generation of active species such as •O_2_ or •O_2_^–^ and then inhibited the degradation rate. An effective hole-capturer, ammonium oxalate (AO), was used to identify the activity of photo-generated holes in the photocatalysis system [53]. In our cases of photodegrading MB, the degrading rate of MB was decreased to 74% when AO was added into the reaction solution. The decrease of the amount of holes also influenced the degradation rate. Both above results might support the argument that the photogenerated hole and electron are all important species for the degrading MB.

In a general case, some radical species with strong oxidation capabilities, such as •OH and •O_2_^–^ species, play an important role for the degradation of pollutants. The •OH is usually generated via the direct photogenerated hole oxidation on the surface of Sm-C-TiO_2_ (OH^−^ + h^+^→•OH or H_2_O + h^+^→•OH + H^+^) or electron-induced multistep reduction of O_2_ (O_2_ + e→•O_2_, •O_2_ + e + 2H^+^→H_2_O_2_, H_2_O_2_ + e→•OH + OH^−^) [54]. The radicals are very reactive and quickly oxidize organic species at the surface of TiO_2_ particles. Tert-Butylalcohol (TBA) was chosen as an •OH scavenger because it reacts with radicals with a high rate constant (*k* = 6 × 10^8^ M^−1^·s^−1^) [55]. As shown in Figure 7, an apparent rate change for MB photodegradation was observed in the presence of TBA in which the degradation rate is decreased to 76% after 6 h irradiation. This result might support the contention that the •OH is also the main oxidation species for the degrading MB. Furthermore, the participation of •O_2_^–^ has been reported to be involved in the photodegradation process [56]. In order to determine its participation in the process, 2 mL of parabenzoquinone (BQ) was introduced to the reaction system. Compared with the scavenger-free system, the presence of the BQ had only a slight effect on the photocatalytic activity and the MB degradation remains almost unaffected. This indicated that the superoxide radical (•O_2_^–^) is a minor factor for degrading MB over Sm-C-TiO_2_.

## 3. Discussion

**Figure 8:**
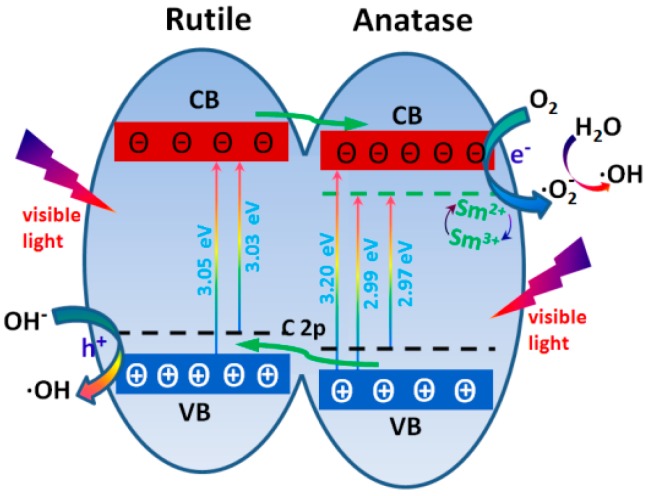
Schematic illustration for the twin synergistic effects of Sm-C co-doping and the mixed phase TiO_2_. In our experiment, the band gap of rutile TiO_2_ may show the band gap of TiO_2_ as the 3.05 eV, the band gap of C-TiO_2_, Sm-TiO_2_, and Sm-C-TiO_2_ are 3.03 eV, 2.99 eV, and 2.97 eV, respectively, indicating that the C and Sm form impurity energy levels.

Based on the above results, it is clear that we have rationally designed and realized the double synergistic effects of Sm-C co-doping and the mixed phase in TiO_2_, considering the synergistic effect of samarium and carbon ions doped into the TiO_2_ samples, optimized light absorption, and surface charge carrier separation/transfer. The XPS results demonstrated that the Sm species doped in photocatalysts exist in the form of Sm^3+^ and Sm^2+^. The empty orbital of Sm^3+^ might trap the photoexcited electrons to produce Sm^2+^. Because of the high activity of Sm^2+^, the electrons can be easily detrapped and transferred to dye adsorbed on the catalyst surface to degrade the dye into a small molecule. Moreover, the electron can also reduce the adsorption of oxygen and then form •OH radicals, suggesting that the Sm^3+^ doping inhibited the recombination of the photogenerated electron and hole [10,17]. On the other hand, carbon doping can be used to raise the valence band (VB) maximum (Figure 8) and it will form a narrower band gap than that of the undoped TiO_2_. This extended the absorption into the visible light region. Under visible light irradiation, photons from visible irradiation were utilized to generate electrons and holes. The electrons exited from the C 2p orbit to the conduction band, the O_2_ adsorbed on the surface captured the electrons to form •O_2_^−^, and the •O_2_^−^ reacted with H_2_O subsequently to form •OH radicals which will react with the organic dye.

In addition, the synergetic effect between anatase and rutile is another reason for the high photocatalytic activity. It has been reported that the energetic alignment exists in the band edges between both TiO_2_ phases with anatase possessing the higher electron affinity and work function [19,24]. Upon combining these two phases, a staggered band gap is formed and the synergistic effect causes an efficient charge separation across the phase junctions.

Therefore, the possible photodegradation mechanism of the as-prepared Sm and C co-doped TiO_2_ is shown in Figure 8. On the basis of the above analysis, the mixed phase TiO_2_ with co-doped Sm and C exhibited improved valence band edges and weakened conductance band edges, which contributed to the enhancement of visible light absorption. That is, the photoexcited and separated electrons from the valence band of the as-prepared catalyst will further transfer into the internal band of the catalyst, which was formed by the Sm^3+^ and Sm^2+^ species under visible light irradiation. Additionally, the effective separation of charge carriers may be another reasonable explanation for the enhancement of photocatalytic activity, because such carriers can transfer between the anatase and rutile phase.

## 4. Materials and Methods

All reagents were of analytical grade supplied by Sigma-Aldrich Co. (St. Louis, MS, USA) and were used as received without further purification. The Sm/C-TiO_2_ was synthesized by a facile sol-gel method. Typically, 5 mL Tetra-n-butyl Titanate (Ti(OC_4_H_9_)_4_) was slowly (1 drop/s) added into 70 mL distilled water containing 3 mL homemade hydrolysis inhibitor under strong stirring at 60 °C, and the milky suspension was adjusted with 2.5 mol/L HNO_3_ until pH = 2. After 0.1455 g glucose (C_6_H_12_O_6_·H_2_O) and 0.0653 g samarium nitrate (Sm(NO_3_)_3_·6H_2_O) was dissolved completely in 10 mL of distilled water, it was added to the Ti(OC_4_H_9_)_4_ solution and the solution was strongly stirred for 16 h to form the sol. The sols were aged for 2 days, dried at 80 °C in air, grinded, and then sintered in air for 3 h at 500 °C. The Sm-C-TiO_2_ catalysts were eventually obtained (Sm/Ti = 1.0% and C/Ti = 30%). The pure TiO_2_, Sm-TiO_2_ (Sm/Ti = 1.0%), and C-TiO_2_ (C/Ti = 30%) powder were also prepared in accordance with a similar process to that mentioned above.

The X-ray diffraction (XRD) patterns with a range of 10°–90° (2θ), were obtained with a D/max-3BX diffractometer (Rigaku, Tokyo, Japan) using Cu Kα radiation (λ = 0.15418) source at 40 kV and 30 mA. The average crystallite size values *(d)* were obtained for the predominant phase of each sample by using the major peak of the patterns, that is, the (110) and (101) planes for the rutile and anatase phases, respectively. The interplanar spacings were calculated by using Bragg’s Law from the diffraction peaks of (110) and (101). The particle sizes of the samples were collected using a Nano-series Zeta Sizer (Malvern, Worcestershire, UK). FT-IR spectra of the samples were recorded using a FTS-40 (BIO-RAD, Berkeley, CA, USA) fourier transform-infrared spectrometer. The X-ray photoelectron spectroscopy (XPS) results of the samples were carried out on a PHI-5500 (Ulvac-PHI Company, Kanagawa, Japan) X-ray photoelectron spectrometer with an Al Kα excitation source (λ = 8.4 Å), and the binding energies were calibrated to the C 1s peak by 284.6 eV. The morphology of Sm-C-TiO_2_ was observed using a transmission electron microscope (TEM, JEM-2100, JEOL Ltd., Tokyo, Japan) operating at an accelerating voltage of 200 kV. SEM and EDX were performed on a scanning electron microscope (Hitachi S-4800 II, Tokyo, Japan) operated at an acceleration voltage of 10 kV to characterize the morphologies and the compositions of the prepared samples. The optical absorbance spectra of the samples were measured on a UV-Vis spectrophotometer (UV-2401PC, Shimadzu, Kyoto, Japan), with BaSO_4_ used as a reference standard. For understanding the recombination of the photogenerated electron and hole pairs of all the samples, the photoluminescence emission (PL) spectra were obtained by using a FL4500 fluorescence spectrophotometer (Shimadzu, Kyoto, Japan).

The methylene blue (MB) dye was chosen as the degrading pollution to test the photocatalytic activities of the as-prepared samples. The photocatalytic reaction was performed in a Pyrex reactor. 0.1 g of the catalyst was dispersed in 50 mL of 10 mg/L MB aqueous solution. The light irradiation system contained a 30 W fluorescent lamp. Prior to irradiation, the suspension was stirred in dark conditions for 45 min to ensure complete adsorption-desorption equilibrium. The degradation efficiencies of MB were evaluated by monitoring the maximum absorption value of the MB chromophoric group at λ_max_ = 664 nm of the MB aqueous solution which was measured by using the VIS7220N UV-Vis spectrophotometer (Beifen-Ruili Analytical Instrument Ltd., Beijing, China). During the irradiation, about 4 mL of the suspension was continually taken from the reaction cell at given time intervals for subsequent target dye concentration analysis after centrifuging at 3500 r/min in a centrifuge for 40 min dispersion. The degradation efficiency was determined by dividing (*C*_0_ − *C*)/*C*_0_, where *C* is the present MB concentration and *C*_0_ is the initial concentration of MB. The concentration of MB is calculated by a calibration curve.

The kinetics of the MB photodegradation on the TiO_2_ surface can be described by the first order reaction kinetics equation:

ln(*C*_0_/*C*) = *kt* (2)

where *C*_0_ is the initial concentration of MB and *C* is the actual concentration of MB at light irradiation time *t*. First-order apparent rate constants (*k*) were calculated by the linear fitting of the experimental data, and used to judge and compare the photocatalytic activity of the catalysts.

## 5. Conclusions

The crystalline anatase/rutile mixed phase Sm-C-TiO_2_ catalysts were successfully prepared by a simple sol-gel method and characterized by different techniques. Samarium acts as an anatase-rutile transformation inhibitor and also favors decreased TiO_2_ grain size, while carbon has the opposite effect. Importantly, both the dopants narrow the band gap energy of TiO_2_. In addition, the synergistic effects of the anatase-rutile heterojunction structure contribute to the enhanced photocatalytic activity. Compared with P25, the light absorption edge of Sm-C-TiO_2_ is red-shifted about 20 nm. High photocatalytic activities were investigated by MB degradation under irradiation from a weak fluorescent lamp. The degradation rate of MB over the Sm-C-TiO_2_ sample was the best; the first-order apparent rate constants increased by about 4.3 and 3.8 times than that of commercial Degusssa P25 and pure TiO_2_ under the same experimental conditions. The MB oxidation was driven mainly by the participation of electrons, holes, and •OH radicals.

## Figures and Tables

**Table 1:** XRD results of the different TiO_2_ samples calcined at 500 °C.

Samples	Anatase *X*_A_ (%) ^a^	Crystal Size		Interplanar Spacing		Average Grain Size (nm)
		*D*_A_/nm	*D*_R_/nm	d-(101) Å	d-(110) Å	
Pure TiO_2_	12.6	29.8	31.8	3.5145	3.2478	174
C-TiO_2_	4.0	31.7	30.5	3.5148	3.2475	143
Sm-TiO_2_	74.3	12.1	22.0	3.5175	3.2525	58
Sm-C-TiO_2_	70.0	11.3	25.9	3.5092	3.2550	78

^a^ Anatase/Rutile proportions were calculated from Spurr and Myers’ equation: *W*_R_/*W*_A_ = 1.22(*I*_R_/*I*_A_) − 0.025 based on the X-ray diffraction patterns [29]. *W*_R_/*W*_A_ stands for the ratio of rutile and anatase phases. *I*_R_ refers to the intensity of the rutile (110) diffraction line, and *I*_A_ refers to the intensity of the anatase (101) diffraction line. The infinity symbol is used for the pure anatase phase or pure rutile phase calculation.
